# Hereditary Angioedema: The Clinical Picture of Excessive Contact Activation

**DOI:** 10.1055/s-0042-1758820

**Published:** 2022-11-23

**Authors:** Remy S. Petersen, Lauré M. Fijen, Marcel Levi, Danny M. Cohn

**Affiliations:** 1 Department of Vascular Medicine, University of Amsterdam, Amsterdam Cardiovascular Sciences, Amsterdam UMC, Amsterdam, the Netherlands

**Keywords:** hereditary angioedema, bradykinin, C1-inhibitor, contact activation, therapy

## Abstract

Hereditary angioedema is a rare, genetic disorder characterized by painful, debilitating and potentially life-threatening angioedema attacks in subcutaneous and submucosal tissue. While usually unpredictable, attacks can be provoked by a variety of triggers including physical injury and certain medication and are often preceded by prodromal symptoms. Hereditary angioedema has a profound influence on the patients' lives. The fundamental cause of hereditary angioedema in almost all patients is a mutation in the *SERPING1* gene leading to a deficiency in C1-inhibitor. Subsequently, the contact activation cascade and kallikrein-kinin pathway are insufficiently inhibited, resulting in excessive bradykinin production triggering vascular leakage. While C1-inhibitor is an important regulator of the intrinsic coagulation pathway, fibrinolytic system and complement cascade, patients do not have an increased risk of coagulopathy, autoimmune conditions or immunodeficiency disorders. Hereditary angioedema is diagnosed based on C1-inhibitor level and function. Genetic analysis is only required in rare cases where hereditary angioedema with normal C1-inhibitor is found. In recent years, new, highly specific therapies have greatly improved disease control and angioedema-related quality of life. This article reviews the clinical picture of hereditary angioedema, the underlying pathophysiology, diagnostic process and currently available as well as investigational therapeutic options.

## Clinical Case

A 21-year-old female was referred to the emergency department with an acute swelling of the throat. She had a history of recurrent episodes of non-pruritic swelling of extremities that usually resolved spontaneously within three days. These episodes started in puberty, occurred about once a month and were never accompanied by hives. The patient had presented to the emergency department twice before, both times with extreme abdominal pain accompanied by vomiting and diarrhea without an identified cause. Her mother had similar episodes of edema, although less frequently. Her grandfather passed away at the age of 30 as a result of asphyxiation. The patient used combined estrogen-progestin oral contraceptives and in the past had tried high-dose anti-histamines without effect on the episodes of swelling. At presentation, epinephrine, clemastine, and dexamethasone, were administered immediately, with no effect on what was diagnosed as laryngeal edema. Laryngeal intubation was necessary to secure the airway and the patient was admitted to the intensive care unit. An empirical dose of plasma-derived C1-inhibitor was administered, after which the edema swiftly resolved. The patient was discharged with a subcutaneous bradykinin B2 receptor inhibitor to be administered in case of another attack. Several weeks later, laboratory tests confirmed the diagnosis of hereditary angioedema (HAE) type 1, due to deficiency of C1-inhibitor. The patient started with prophylactic subcutaneous plasma-derived C1-inhibitor concentrates twice weekly and switched to a type of contraception without estrogen. In the following months, she continued to experience occasional break-through attacks that responded well to self-administered subcutaneous bradykinin B2 receptor inhibitor.

## Clinical Features

HAE is a rare, genetic disease that is estimated to affect about 1 in 50,000 to 100,000 individuals. 1 Patients with HAE have recurring episodes of non-pitting, non-pruritic angioedema. Angioedema occurs in subcutaneous and submucosal areas and causes different symptoms depending on the location. If untreated, attacks typically worsen in the first 24 hours before resolving in two to five days. 2 Contrary to mast cell-mediated angioedema, attacks are not accompanied by urticaria or anaphylaxis. 2 Onset of symptoms is usually during childhood or adolescence, with a mean age at first symptoms of 11 years. 3 Frequency and locations of attacks vary significantly between patients and can even vary during a single patient's lifetime. 2 Up to 30% of patients experience edema at multiple anatomically unrelated regions simultaneously. 4 Hindering and debilitating dermal swelling predominantly occurs in the extremities, in the orofacial or genital region. When submucosa of the gastrointestinal tract is involved, patients present with extreme abdominal pain and vomiting, mimicking an acute abdomen, in some cases leading to unnecessary surgical interventions. HAE patients are estimated to be two-and-a-half times more likely to undergo abdominal surgery compared to the general population. 5 In the case of upper airway edema, impending airway obstruction can lead to asphyxiation if the airway is not secured or the edema treated in time. A review of literature published in 1962, before adequate treatment was developed, reported that 77% of HAE patients died due to acute laryngeal edema. 6 More recently, worldwide mortality amongst HAE patients due to laryngeal edema was still estimated to be as high as 5%. 7

**Fig. 1:**
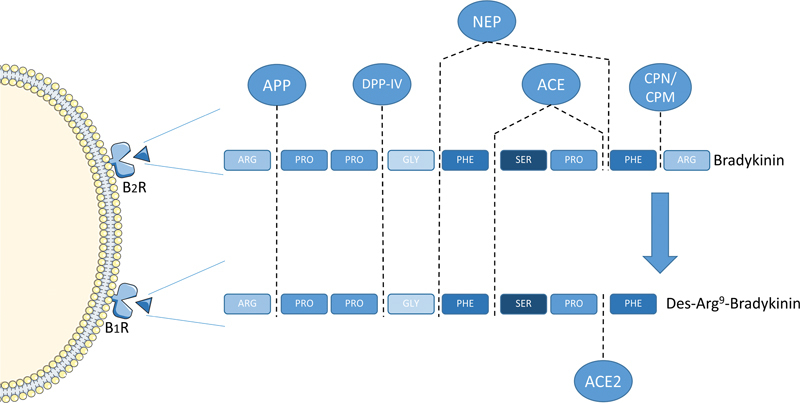
Degradation of bradykinin. Bradykinin binds to the bradykinin B2 receptor (B2R) and is transformed to des-arg9-bradykinin by carboxypeptidase N and M (CPN/CPM), which in turn binds to the bradykinin B1 receptor (B1R). Bradykinin is principally degraded by angiotensin converting enzyme (ACE), while angiotensin converting enzyme 2 (ACE2) has a higher affinity for des-arg9-bradykinin. Both bradykinin and des-arg9-bradykinin are broken down into inactive parts by aminopeptidase P (APP), dipeptidyl peptidase IV (DPP-IV), and neutral endopeptidase (NEP).

Various events and circumstances can trigger attacks of angioedema. Trauma caused by invasive procedures is well known to induce attacks and therefore pre-procedural prophylactic medication is recommended. 8 Patients should refrain from inhibitors of angiotensin-converting enzyme or dipeptidyl peptidase IV for inhibition of these enzymes is known to increase attack frequency as illustrated in Fig. 1 . 8 Increased exogenous or endogenous estrogen levels typically enhance attacks in female patients. This can occur in physiological circumstances like pregnancy and during the menstrual cycle, and also with the use of oral contraceptives or hormonal replacement therapy. 9 Other reported triggers include infection, fever, mental stress, and physical activity. 10 11

The occurrence of angioedema attacks is often unpredictable; however, 90% of patients experience prodromal symptoms hours to days before an attack. 12 13 14 These symptoms span a wide range including dermal manifestations, tiredness, emotional disturbances and gastro-intestinal symptoms. The most common dermal manifestation described to precede HAE attacks is erythema marginatum. This non-pruritic skin rash is sometimes misinterpreted as urticaria and is associated with a longer diagnostic delay. 12 The majority of patients report to be able to predict the onset of an attack and a recent study described the sensitivity and specificity of prodromes as a predictor of attacks as 89.5 and 63.1%, respectively. 14

Frequent unpredictable and debilitating angioedema attacks can have a significant impact on the daily life of HAE patients. Depression and anxiety are reported in a substantial percentage of HAE patients, corresponding with a lower than average quality of life with regard to mental health and social relationships. 15 16 17 18 Patients report a loss of productivity and absence from work due to attacks. Especially facial, abdominal, and laryngeal attacks correlate with higher absenteeism and activity impairment. 17 Even in attack-free periods, patients report anxiety due to the unpredictability of attacks, as well as worries about passing HAE on to their offspring. 18 HAE is shown to influence reproductive choices in approximately 20% of HAE patients. 19

## Pathophysiology

**Fig. 2:**
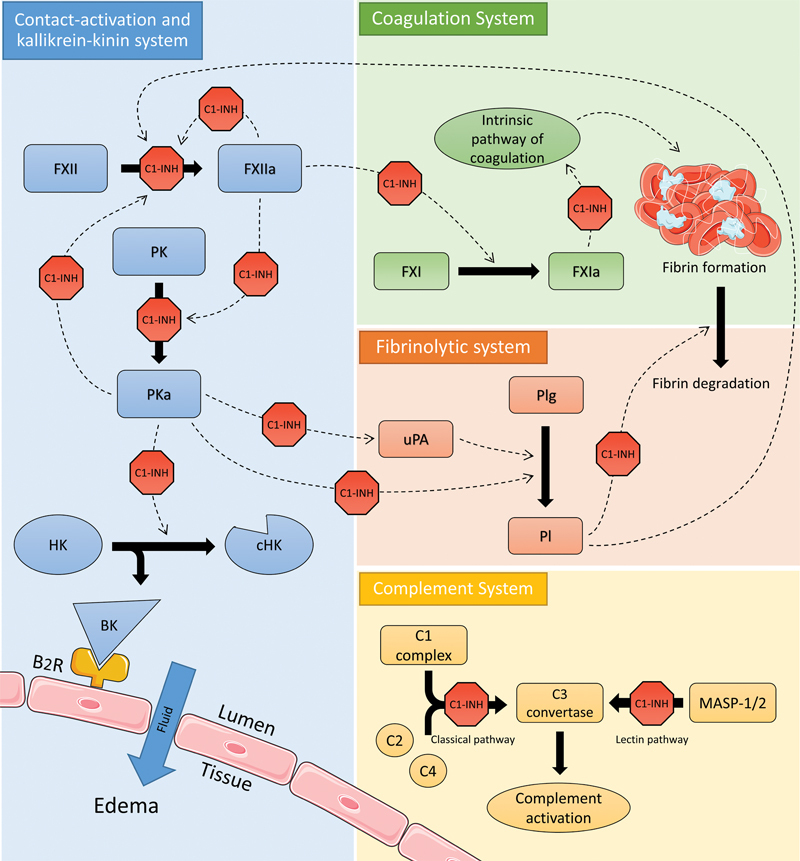
Functions of C1-inhibitor. C1-inhibitor (C1-INH) is a crucial regulator of several intravascular cascades, including the contact-activation system and kallikrein-kinin pathway. The contact-activation system starts when factor XII (FXII) is converted in active factor XII (FXIIa), illustrated by a solid arrow. The activation of plasma prekallikrein (PK) in plasma kallikrein (PKa) is subsequently stimulated by FXIIa, as illustrated by a dotted arrow. Kallikrein, in turn, induces further activation of FXII and facilitates the cleavage of high-molecular weight kininogen (HK) into cleaved high-molecular weight kininogen (cHK) and bradykinin, which in turn binds to the bradykinin B2 receptor (B2R), resulting in the efflux of fluid to extravasal tissue. In the coagulation system, C1-INH inhibits both the activation of factor XI (FXI) and the function of active FXI (FXIa). In the fibrinolytic pathway, kallikrein induces the activation of plasminogen (Plg) to plasmin (Pl) directly and indirectly through the stimulation of urokinase plasminogen activator (uPA). C1-INH inhibits these functions of kallikrein as well as the direct effects of plasmin on fibrin degradation. In the complement system, both the classical pathway, involving complement 1 complex (C1 complex), complement 2 (C2), and complement 4 (C4, as well as the lectin pathway involving mannose-associated serine protease 1 and 2 (MASP-1/2) are inhibited by C1-INH.

In HAE patients, angioedema is caused by insufficient inhibition of the contact activation pathway and kallikrein-kinin system. In the majority of patients, this is caused by an absolute C1-inhibitor deficiency (type 1, 85% of HAE with C1-inhibitor deficiency) or C1-inhibitor dysfunction (type 2, 15% of HAE with C1-inhibitor deficiency). C1-inhibitor is a heavily glycosylated single chain polypeptide of 478 amino acid residues with a molecular weight of 105 kDa. The physiological plasma concentration is about 240 mg/L with a long estimated half-life of about 70 hours. 20 C1-inhibitor regulates various pathways, including the kallikrein-kinin system, intrinsic coagulation system, fibrinolytic pathway, and the complement cascade ( Fig. 2 ). The contact activation route and subsequent kallikrein-kinin system are initiated when factor XII (FXII) is autoactivated upon contact with negatively charged surfaces and subsequently induces cleavage of the zymogen prekallikrein into plasma kallikrein (PKa). PKa activates more FXII in a positive feedback loop and induces cleavage of high-molecular-weight kininogen, thereby producing bradykinin, 21 a nonapeptide of the kinin family. Bradykinin is considered to be principally responsible for the vasodilation and subsequent transfer of fluid to extravasal tissue in the case of angioedema formation in HAE patients. Bradykinin binds to the bradykinin B2 receptor (BKB2R) expressed on endothelial cells, initiating a signaling cascade leading to increased vascular permeability through several mechanisms including the release of nitric oxide, contraction of endothelial cells, and the uncoupling of tight junctions between endothelial cells. Carboxypeptidases N and M cleave the terminal arginine from bradykinin, forming des-Arg9-bradykinin, an agonist for the bradykinin B1 receptor (BKB1R). 21 This receptor is not constitutively present on endothelial cells, but is expressed following tissue injury or inflammatory stimuli. It is unclear what the function of the BKB1R is in the formation of angioedema in the context of HAE. The receptor is slowly and partially desensitized after binding, in contrast to the BKB2R which is rapidly desensitized. 22 Therefore, it has been hypothesized that stimulation of the BKB1R could explain the long duration of angioedema attacks. 23 Bradykinin and des-Arg9bradykinin are rapidly broken down into inactive metabolites by aminopeptidase P, neutral endopeptidase, dipeptidyl peptidase IV, and angiotensin-converting enzyme. 24

C1-inhibitor is an important regulator of other pathways as well. C1-inhibitor regulates the intrinsic coagulation pathway by partially inhibiting the activation of factor XI and the functions of activated factor XI. 25 Likewise, C1-inhibitor regulates the fibrinolytic system directly by inhibiting plasmin activity, though less efficient than α2-antiplasmin, 26 and indirectly by inhibiting the conversion of plasminogen to plasmin by PKa. 20 Signs of activation of the thrombotic and fibrinolytic systems in HAE patients include elevated levels of activated factor XII (FXIIa), D-dimer, plasmin-α2-antiplasmin complexes and thrombin generation during attacks. 27 28 29 30 Despite the impact of C1-inhibitor on both coagulation and fibrinolysis, C1-inhibitor deficiency does not translate into an increased risk of thrombosis or bleeding. 31 Furthermore, C1-inhibitor is a key player in the complement system, for it is the only known inhibitor of C1r and C1s proteases in the classical complement pathway. It also regulates the lectin pathway by inactivating mannose-associated serine proteases 1 and 2. 32 33 There is no proven association between immunodeficiency disorders or autoimmune conditions and HAE. 34

In HAE type 1 and 2, lack of (functional) C1-inhibitor is caused by a mutation in the *SERPING1* gene. About 750 different pathogenic mutations are reported to be associated with HAE type I, including missense, nonsense, deletions, insertions, duplications and splice site mutations. 35 36 Most cases of HAE type 2 are caused by a missense mutation in exon 8, resulting in the reduced ability to inhibit target proteases. 22 HAE is inherited as an autosomal dominant trait in almost all cases. While HAE type I patients possess one functional allele, levels of C1-inhibitor normally vary between 5 and 30% of normal levels. Recent studies suggest that in a limited subset of mutations, this could be due to the formation of polymers between functional and dysfunctional C1-inhibitor leading to intracellular retention. 37 38 Homozygous forms of HAE have long been considered embryonically lethal and only in recent years case reports of consanguineous families with homozygous HAE have sporadically been published. The inheritance pattern in these families is most likely recessive since heterogeneous members of these families are almost always asymptomatic. An unusual biochemical feature found in some patients with homozygous C1-INH deficiency is a significant reduction in C1q levels. 39 40 41 42

In rare cases, HAE with normal functional and absolute levels of C1-inhibitor (HAE-nC1-INH) occurs. This type of HAE has a very heterogeneous phenotype. Known mutations causing this third type of HAE are summarized in Table 1 . In up to 25% of HAE-nC1-INH patients a mutation in the gene coding for FXII (HAE-FXII) is found, leading to an increase in autoactivation of FXII. While this mutation is inherited as an autosomal dominant trait, the majority of symptomatic carriers is female, 43 implying an association with estrogen. In patients with HAE type 1 and 2, the influence of estrogen on attack frequency and severity has been described extensively. 44 Previously suggested mechanisms for this observation are the increase of FXII levels, decrease of C1-inhibitor levels, induction of the release of nitric oxide and impairment of the functionality of angiotensin converting enzyme. 44 45 The specific characteristics of HAE-FXII suggest the influence of estrogen on FXII might be an important factor in the development of angioedema. Another noteworthy difference in the clinical presentation of patients with HAE-FXII is the absence of erythema marginatum as a prodromal symptom. 46

**Table 1:** Mutations associated with HAE-nC1-INH

Affected gene	First reported by	Known mutations	Suspected agent	Suspected mechanism
*F12*	Dewald and Bork 97	T328K 97 T328R 97 c.971_1018 + 24del72 98 c.892_909dup 99	Bradykinin	Increased autoactivation of factor XII or increased sensitivity to activation by plasmin
*ANGPT1*	Bafunno et al 100	A119S A8V Q370H	Vascular endothelium	Disruption of the multimerization of angiopoetin-1, leading to a reduced ability to bind to its receptor on endothelial cells
*PLG*	Bork et al 101	K330E	Bradykinin	Direct liberation of bradykinin from high-molecular weight kininogen 102
*KNG1*	Bork et al 103	M379K	Bradykinin	Mechanism remains unclear
*MYOF*	Ariano et al 104	R217S	Vascular endothelium	Increased activation of VEGF-mediated intracellular signaling
*HS3ST6*	Bork et al 105	T144S	Unclear	Incomplete synthesis of heparan sulfate by heparan sulfate-glucosamine 3- *O* -sulfotransferase, possibly affecting the interaction between endothelial cell surface and high-molecular weight kininogen

ANGPT1, angiopoietin-1 gene; F12, factor XII gene; HAE-nC1-INH, hereditary angioedema with normal C1-inhibitor; HS3ST6, heparan sulfate-glucosamine 3-O-sulfotransferase 6 gene; KNG1, Kininogen-1 gene; MYOF, myoferlin gene; PLG, plasminogen gene; VEGF, vascular endothelial growth factor

## Diagnosis

When HAE is suspected, serum levels of C1-inhibitor protein, C1-inhibitor function, and complement component 4 are measured ( Table 2 ). While HAE type 1 and 2 are clinically indistinguishable, laboratory tests will make it possible to differentiate between these types: serum of patients with type 1 HAE will show a C1-inhibitor protein level of <50% of normal combined with a decreased functionality of C1-inhibitor, whereas patients with type 2 will have a decrease in C1-inhibitor functionality in the presence of normal or increased levels of C1-inhibitor protein. Even though C4 levels are usually decreased in HAE patients, it is not recommended to use this as a single screening tool due to its limited sensitivity and specificity, estimated at 81 and 85%, respectively. 47 The most recent guideline recommends to repeat C1-inhibitor function, C1-inhibitor protein, and C4 testing in patients to confirm the diagnosis. 8 Genetic sequencing is not recommended as a diagnostic tool for patients with HAE type 1 or 2. However, in case of a high suspicion of HAE with normal C1-inhibitor, assessment for known mutations underlying HAE-nC1-INH is recommended. 8 While C1-inhibitor level and function remain the golden standard for diagnosis of HAE type 1 and 2, analysis can take weeks, making them impractical to distinguish HAE from other forms of angioedema in the acute setting. In patients that are already diagnosed with HAE, there is a need for biomarkers to correctly identify and predict attacks. While recent studies have yielded promising results, none are recommended to be used in daily practice yet. 48 49 A reduced level of C1-inhibitor protein has a short differential diagnosis. However, if a patient with laboratory findings corresponding with HAE type 1 has a negative family history and onset of symptoms later in life (>30 years), it is important to exclude acquired angioedema with C1-inhibitor deficiency (AAE-C1-INH). This condition has an even lower prevalence than HAE and is estimated to affect 1 in 500,000 individuals. It is associated with plasma cell dyscrasias, lymphoproliferative disorders, and autoimmune diseases that cause a deficiency of C1-inhibitor either through neutralizing antibodies or through increased consumption by massive activation of the complement system. 50 51 Contrary to HAE, laboratory tests of about 75% of AAE-C1-INH patients show low levels of C1q and in part of the population C1-inhibitor autoantibodies can be identified. 52 53 AAE-C1-INH requires additional diagnostics to exclude or confirm associated disorders and has different treatment options than HAE, therefore it is important to distinguish between these disorders. 54

**Table 2:** Laboratory findings in HAE and AAE-C1-INH

	C1-INH level	C1-INH function	C4 level	C1q level
HAE Type 1	↓	↓	↓	N
HAE Type 2	N/↑	↓	↓	N
HAE-nC1-INH	N	N	N	N
AAE-C1-INH	↓	↓	↓	N/↓

Abbreviations: AAE-C1-INH, acquired angioedema with C1-inhibitor deficiency; C1-INH, C1-inhibitor; HAE, hereditary angioedema; HAE-nC1-INH, hereditary angioedema with normal C1-inhibitor; N, normal

HAE patients have a 50% chance to pass the affected gene on to their offspring. However, severity and frequency of symptoms are highly variable even within one family. Notably, in about 25% of patients a *de novo* mutation occurs. 55 Recent guidelines recommend to test children from HAE patients as early as possible. 8 56 However, the testing of neonates and infants proves difficult, since antigenic and functional C1-inhibitor levels in peripheral and cord blood of healthy children are lower than in adults. 57 Therefore, tests performed in infants should be repeated after the child is one year old. Genetic testing (preferably of umbilical cord blood) can be considered if the mutation of the parent is known, which is not the case in up to 10% of patients. 8 56 Prenatal diagnosis and pre-implantation genetic testing are available depending on national laws and practice. 9 19

## Treatment

In contrast to mast cell-mediated forms of angioedema, HAE attacks do not respond to epinephrine, antihistamines, or corticosteroids. 58 In recent years, new improved therapies have drastically changed the landscape of HAE and have even led to a change in treatment goals. In 2021, a global Delphi initiative among HAE specialists concluded that the ultimate goals of treatment should be complete control of the disease and normalization of the patient's life. 59 More highly effective drugs are expected to become available in the near future, making the ambitious treatment goals more realistic for a growing number of patients. Currently available treatment options and therapies in development are summarized in Table 3 .

**Table 3:** Treatment options for HAE currently available or in development

Drug	Manufacturer (Trade name)	Indication	Mechanism of action	Administration	Regulatory / developmental status
Currently available treatment					
Attenuated androgens (danazol, oxandrolone, stanozolol)	Generic manufacturers	LTP, STP	Induction of APP activity, increased C1-INH synthesis and C1-INH mRNA expression	Oral	Licensed
Berotralstat	BioCryst (Orladeyo)	LTP	Small molecule plasma kallikrein inhibitor	Oral	Licensed
Conestat alfa	Pharming (Ruconest)	ODT	Recombinant C1-INH concentrate replacing deficient C1-INH	Intravenous	Licensed
Ecallantide	Takeda (Kalbitor)	ODT	Plasma kallikrein inhibitor	Subcutaneous	Licensed only in United States
Icatibant	Takeda (Firazyr)	ODT	Bradykinin B2 receptor antagonist	Subcutaneous	Licensed
Lanadelumab	Takeda (Takhzyro)	LTP	Monoclonal antibody plasma kallikrein inhibitor	Subcutaneous	Licensed
Plasma-derived C1-INH concentrate	CSL Behring (Berinert), CSL Behring (Haegarda) Takeda (Cinryze)	ODT, LTP, STP	Replacement of deficient C1-INH	Intravenous or subcutaneous	Licensed
Developmental treatment					
ATN-249	Attune Pharmaceuticals	LTP	Small molecule plasma kallikrein inhibitor	Oral	Phase I
BMN 331	BioMarin	Curation	AAV5-based gene therapy of C1-INH	Intravenous	Phase 1
Donidalorsen	Ionis	LTP	Antisense oligonucleotide targeted at prekallikrein	Subcutaneous	Phase 3
Garadacimab	CSL Behring	LTP	Monoclonal antibody FXIIa inhibitor	Subcutaneous	Phase 3
KV998086	KalVista	LTP	Small molecule FXIIa inhibitor	Oral	Preclinical
NTLA-2002	Intellia	Curation	CRISPR-CAS9-based gene editing of prekallikrein	Intravenous	Phase 1
OTL-105	Orchard Therapeutics and Pharming Group	Curation	Ex vivo autologous hematopoietic stem cell gene therapy to restore C1-INH production	Intravenous	Preclinical
PHA-022121	Pharvaris	LTP and ODT	Bradykinin B2 receptor antagonist	Oral	Phase 2
Sebetralstat	KalVista	ODT	Small molecule plasma kallikrein inhibitor	Oral	Phase 3
STAR-0215	Astria Therapeutics inc.	LTP	Monoclonal antibody plasma kallikrein inhibitor	Subcutaneous	Preclinical
Unnamed	Spark Therapeutics	Curation	Liver-directed gene therapy	Intravenous	Preclinical
Unnamed	RegenxBio	Curation	AAV-based gene therapy	Intravenous	Preclinical

Abbreviations: AAV, adeno-associated virus; APP, aminopeptidase P; C1-INH, C1-inhibitor; CRISPR-CAS9, clustered regularly interspaced short palindromic repeats and CRISPR-associated protein 9; FXIIa, active Factor XII; HAE, hereditary angioedema; LTP, long-term prophylaxis; mRNA, messenger ribonucleic acid; ODT, on-demand treatment; STP, short-term prophylaxis

HAE treatment consists of a twin-track approach, comprising of symptom relief in the case of an attack on one hand, and the overall decrease in attacks on the other hand. The most recent guideline advises to treat an HAE attack with on demand treatment (ODT) as early as possible, for this is associated with the best treatment response. 60 61 62 First line ODT options are plasma-derived or recombinant C1-inhibitor, icatibant or ecallantide. 8 Plasma-derived C1-inhibitor was first administered to an HAE patient in 1979 63 and was approved by the Food and Drug Administration in 2008. Self-administration of intravenous plasma-derived C1-inhibitor concentrate is shown to be safe and effective, 64 though complications may arise if frequent venous access is required. 65 Even though C1-inhibitor has a direct effect on both the intrinsic coagulation and the fibrinolytic pathway, there is no evidence of an elevated risk of thromboembolic events with the use of C1-inhibitor concentrate at recommended doses. 66 A recombinant intravenous C1-inhibitor concentrate called conestat alfa was developed as an alternative for plasma-derived C1-inhibitor. 67 Another frequently used ODT option is icatibant, a competitive selective antagonist of the BKB2R. 68 Icatibant can be self-administered subcutaneously and has been proven to efficiently shorten the time to onset of symptom relief, especially when administered quickly after the first symptoms have emerged. 60 68 In the United States of America, the subcutaneous direct PKa inhibitor ecallantide is available for ODT. Efficacy has been proven, 69 however, utilization is limited for it can only be administrated by a health care professional with adequate medical support due to the occurrence of anaphylaxis in 3.5% of patients. 70 Before the development of specific therapies, fresh-frozen plasma has been used to replenish deficient C1-inhibitor in case of severe, life-threatening attacks, although not without risks. 71 72 Nowadays, the use of fresh-frozen plasma is only recommended if no targeted therapies are available, 8 which is still the case in some countries where targeted therapies are unaffordable or not yet registered. 73

Short-term prophylaxis (STP) is indicated for invasive procedures known to trigger attacks. The only recommended first-line STP in the most recent guideline is intravenous plasma-derived C1-inhibitor. 8 The need for long-term prophylaxis (LTP) is patient-specific and depends on disease activity and burden, as well as patient preferences. 8 The recently updated guideline advises the following first line options for LTP: plasma-derived C1-inhibitor, lanadelumab, and berotralstat. 8 A new subcutaneous form of plasma-derived C1-inhibitor demonstrated a positive effect on HAE-related quality of life. 74 75 In recent years, two new prophylactic therapies targeting PKa have been licensed by the Food and Drug Administration and the European Medicines Agency. Lanadelumab is an IgG1 type monoclonal antibody directed at PKa with a high efficacy: a subcutaneous dose once every 2 weeks reduced angioedema attacks with more than 90% in a placebo-controlled trail. 76 Berotralstat is an oral PKa inhibitor that reduced angioedema attacks by 44% compared to placebo in a phase 3 study. 77 Attenuated androgens (danazol, oxandrolone, and stanozol) have been used as first-line STP and LTP for more than four decades. 78 They are thought to reduce attack frequency through various mechanisms: induction of C1-inhibitor synthesis in hepatocytes, increased expression of C1-inhibitor mRNA in peripheral blood mononuclear cells, 79 and increased bradykinin degradation by induction of aminopeptidase P activity. 80 While effective in some, long-term use of attenuated androgens comes with considerable side effects in a majority of patients. 81 With the development of effective alternatives, attenuated androgens are now only recommended as second-line LTP. 8 Likewise, antifibrinolytics such as tranexamic acid have long been used as LPT as they prevent plasmin-mediated amplification of FXII activation by inhibiting the activation of plasminogen into plasmin. However, due to insufficient efficacy, they are no longer recommended in the latest international guideline. 8

New targeted therapies now in development are highly specific to individual components of the contact activation pathway or kallikrein-kinin system. Therapies targeting FXIIa prevent angioedema by hampering contact activation at the very beginning of the cascade. Garadacimab is a IgG4 type recombinant monoclonal antibody directed against FXIIa that is administered subcutaneously. It showed an almost 99% reduction in breakthrough attacks compared to placebo in a phase 2 trial. 82 KalVista has announced to anticipate the start of clinical studies with KV998086, a prophylactic oral FXIIa inhibitor in 2023. 83 84 Other investigational drugs target PKa or its zymogen prekallikrein (PK), thereby directly preventing the cleavage of high-molecular-weight kininogen and liberation of bradykinin as well as haltering the acceleration of the kallikrein-kinin cascade by preventing the activation of more FXII by kallikrein. Donidalorsen is an antisense oligonucleotide targeted at PK that showed a significant reduction in attack rate of 90% and improvement in angioedema-related quality of life compared to placebo. 85 86 Notably, the reduction in PKa level does not lead to changes in thrombin formation or fibrinolytic activity. 87 Investigational drugs that target PKa include sebetralstat, 88 89 ATN-249 91 and STAR-0215. 92 Therapies targeting the BKB2R prevent angioedema by inhibiting the effects of the end-product of the contact activation cascade and kallikrein-kinin system, bradykinin. Oral BKB2R antagonist PHA-022121 is currently in development for both LTP and ODT. 93

The field of HAE might well be revolutionary changed again in upcoming years, for several gene therapies are currently in development with the goal of curation. One of these is BMN 331, an adeno-associated virus-based gene therapy vector targeting hepatocytes to express more wild-type C1-inhibitor. 94 With NTLA-2002, the Nobel-prize winning CRISPR-Cas9 technique is used to inactivate the *KLKB1* gene in hepatocytes, aiming to continuously suppress HAE activity by reducing the level of PK. 95 These gene therapies are currently studied in phase 1/2 trials in patients. Following a different approach, OTL-105 makes use of *ex vivo* autologous gene therapy to express the *SERPING1* gene in hematopoietic stem cells and is currently studied in pre-clinical trials. 96 Furthermore, RegenxBio and Spark Therapeutics have announced to be in the early stages of development of a gene therapy for HAE.

## Conclusion

While C1-inhibitor is an important regulator in several intravascular pathways, symptoms in HAE patients are caused by insufficient inhibition of the contact activation pathway and kallikrein-kinin system. The subsequent unpredictable and potentially life-threatening angioedema attacks have a pronounced effect on quality of life. However, the future is bright for patients like the 21-year old female in our clinical case. New, highly specific therapies have the potential to drastically reduce their attack rate, improve their safety, and enable them to lead a normal life.
